# Tumor-selective, antigen-independent delivery of a pH sensitive peptide-topoisomerase inhibitor conjugate suppresses tumor growth without systemic toxicity

**DOI:** 10.1093/narcan/zcab021

**Published:** 2021-06-04

**Authors:** Sophia Gayle, Robert Aiello, Nalin Leelatian, Jason M Beckta, Jane Bechtold, Patricia Bourassa, Johanna Csengery, Robert J Maguire, Dan Marshall, Ranjini K Sundaram, Jinny Van Doorn, Kelli Jones, Hunter Moore, Lori Lopresti-Morrow, Timothy Paradis, Laurie Tylaska, Qing Zhang, Hannah Visca, Yana K Reshetnyak, Oleg A Andreev, Donald M Engelman, Peter M Glazer, Ranjit S Bindra, Vishwas M Paralkar

**Affiliations:** Cybrexa Therapeutics, New Haven, CT 06511, USA; Cybrexa Therapeutics, New Haven, CT 06511, USA; Department of Pathology, Yale University School of Medicine, New Haven, CT 06520, USA; Department of Therapeutic Radiology, Yale University School of Medicine, New Haven, CT 06520, USA; Cybrexa Therapeutics, New Haven, CT 06511, USA; Cybrexa Therapeutics, New Haven, CT 06511, USA; Cybrexa Therapeutics, New Haven, CT 06511, USA; Cybrexa Therapeutics, New Haven, CT 06511, USA; Cybrexa Therapeutics, New Haven, CT 06511, USA; Department of Therapeutic Radiology, Yale University School of Medicine, New Haven, CT 06520, USA; Department of Therapeutic Radiology, Yale University School of Medicine, New Haven, CT 06520, USA; Cybrexa Therapeutics, New Haven, CT 06511, USA; Cybrexa Therapeutics, New Haven, CT 06511, USA; Cybrexa Therapeutics, New Haven, CT 06511, USA; Cybrexa Therapeutics, New Haven, CT 06511, USA; Cybrexa Therapeutics, New Haven, CT 06511, USA; Cybrexa Therapeutics, New Haven, CT 06511, USA; Physics Department, University of Rhode Island, Kingston, RI 02881, USA; Physics Department, University of Rhode Island, Kingston, RI 02881, USA; Physics Department, University of Rhode Island, Kingston, RI 02881, USA; Department of Molecular Biophysics and Biochemistry, Yale University, New Haven, CT 06511, USA; Department of Therapeutic Radiology, Yale University School of Medicine, New Haven, CT 06520, USA; Department of Genetics, Yale University School of Medicine, New Haven, CT 06520, USA; Department of Pathology, Yale University School of Medicine, New Haven, CT 06520, USA; Department of Therapeutic Radiology, Yale University School of Medicine, New Haven, CT 06520, USA; Cybrexa Therapeutics, New Haven, CT 06511, USA

## Abstract

Topoisomerase inhibitors are potent DNA damaging agents which are widely used in oncology, and they demonstrate robust synergistic tumor cell killing in combination with DNA repair inhibitors, including poly(ADP)-ribose polymerase (PARP) inhibitors. However, their use has been severely limited by the inability to achieve a favorable therapeutic index due to severe systemic toxicities. Antibody-drug conjugates address this issue via antigen-dependent targeting and delivery of their payloads, but this approach requires specific antigens and yet still suffers from off-target toxicities. There is a high unmet need for a more universal tumor targeting technology to broaden the application of cytotoxic payloads. Acidification of the extracellular milieu arises from metabolic adaptions associated with the Warburg effect in cancer. Here we report the development of a pH-sensitive peptide-drug conjugate to deliver the topoisomerase inhibitor, exatecan, selectively to tumors in an antigen-independent manner. Using this approach, we demonstrate potent *in vivo* cytotoxicity, complete suppression of tumor growth across multiple human tumor models, and synergistic interactions with a PARP inhibitor. These data highlight the identification of a peptide-topoisomerase inhibitor conjugate for cancer therapy that provides a high therapeutic index, and is applicable to all types of human solid tumors in an antigen-independent manner.

## Graphical Abstract

**Graphical Abstract:**
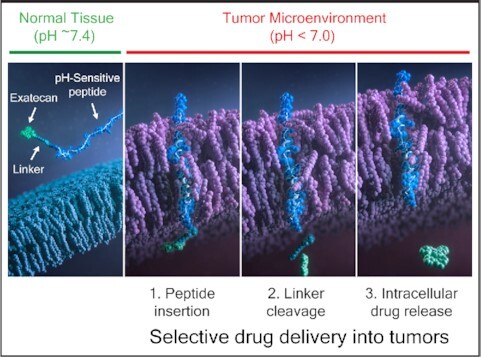
CBX-12 is a pH-sensitive peptide-drug conjugate that selectively delivers the TOP1 inhibitor exatecan into tumor cells in an antigen agnostic manner. The peptide forms an alpha helix only in the low pH tumor microenvironment, allowing directional insertion of the peptide across the tumor membrane, cleavage of the linker within the cytosol, and release of free exatecan within the tumor cell. This pH-based delivery approach results in a high therapeutic index universally across solid tumors.

## INTRODUCTION

Topoisomerase inhibitors are potent DNA damaging agents which are used extensively in oncology to treat a diverse range of cancers (1). Topoisomerases I and II (TOP1/2) are enzymes which regulate DNA structure by cleaving and rejoining DNA during normal cell cycle progression (2). Multiple TOP1 and TOP2 inhibitors have been either Food and Drug Administration (FDA) approved or are in clinical trials for a number of cancers (1,3). These drugs are active as a monotherapies, but they also demonstrate robust synergy when combined with DNA repair inhibitors, including small molecules which target poly(ADP)-ribose polymerase (PARP) and ataxia telangiectasia and Rad3 related (ATR) (4,5).

Exatecan is a derivative of the TOP1 inhibitor camptothecin, with improved potency and robust efficacy across a broad range of tumor models *in vitro* and *in vivo* (6). Exatecan was tested in multiple clinical trials with promising efficacy signals, however dose-limiting toxicities prevented its further development as a systemically administered monotherapy (6). These toxicities included profound myelosuppression and life-threatening gastrointestinal (GI) toxicity (1,7,8).

ADC-based delivery strategies have been developed as an approach to address the unfavorable therapeutic index associated with cytotoxic drugs, including TOP1 inhibitors (1). Trastuzumab deruxtecan (DS-8201a) is a recently FDA-approved ADC which binds to the human epidermal growth factor receptor-2 (HER2) to deliver an exatecan derivative (9). More recently, a Trop-2-directed antibody conjugated to a topoisomerase I inhibitor (SN-38) was FDA-approved for patients with metastatic breast cancer (sacituzumab govitecan) (10). These two examples using ADCs highlight the ongoing interest in developing tumor-targeted topoisomerase inhibitors for the treatment of cancer. However, ADCs are limited to small subsets of tumors expressing high levels of specific antigens, which must have little to no expression in normal tissue for clinical utility (11). Furthermore, side effects caused by linker instability and target antigen expression in healthy tissues are major issues, which likely underlie the persistent dose-limiting toxicities seen with ADCs in the clinic.

It is well established that due to the Warburg effect, the extracellular pH (pH_ex_) in the tumor cell microenvironment is low and ranges between 6.7 to 7.1 with a pH of 6.0 to 6.5 at tumor cell surfaces (pH_surf_) (12). This is in opposition to the extracellular pH of normal cells, which is about 7.4 (12). In contrast to the differences in extracellular pH between healthy and cancerous tissues, intracellular pH exists in a narrow range of 7.2 to 7.4 for all tissues. Thus, the tumor microenvironment is characterized by a ‘reverse pH gradient’ relative to normal cells. We recently developed a peptide-drug conjugate (PDC)-based approach which targets tumor cell surface acidity, a universal feature of cancers, as a means to deliver extremely potent, cytotoxic agents directly to tumors in an antigen-independent manner. These conjugates leverage a variant of a pH-Low Insertion Peptide (pHLIP) recently published by Wyatt *et al.* (13), which senses cell surface acidity and forms a transmembrane alpha helix only in low pH conditions, resulting in directional insertion of the peptide across cancer cell membranes and yielding delivery of C-terminal warheads across the membrane with subsequent intracellular release of the agent via glutathione reduction of the linker (14).

Here, we report the identification and characterization of CBX-12, a highly active conjugate that can deliver high doses of exatecan directly to tumors. We demonstrate robust tumor cell killing across multiple xenograft models *in vivo*, with minimal to no bone marrow and GI toxicity. Notably, we find that efficient tumor cell killing can be achieved with as little as four doses of CBX-12. Finally, we demonstrate that CBX-12 can be safely and efficaciously combined with the FDA-approved oral PARP inhibitor talazoparib (BMN673) to target BRCA1/2-wild-type breast cancers (15). These data highlight the feasibility and potential of antigen-independent and mutation-independent tumor targeting based on low pH at the tumor cell surface, which can be utilized to treat essentially all solid tumor types.

## MATERIALS AND METHODS

### LC–MS/MS Measurement of exatecan plasma concentrations

A 10 mM stock of CBX-12 or exatecan was prepared in DMSO then diluted for an intermediate stock of 100 μM in 1 M Tris pH 7.5 with 1% DMSO. The intermediate stock was used to spike mouse, rat, dog or human plasma, by diluting 1:10 directly in the plasma (300 μl of a 100 μM intermediate stock + 2700 μl plasma) to give a final concentration of 10 μM in plasma containing 0.1% DMSO. Samples were mixed by inversion and a 50 μl time zero sample was collected and immediately frozen at −80°C. Remaining spiked plasma samples were incubated at 37°C and additional 50 μl samples were collected at 2, 4, 8 and 24 h and immediately frozen at −80°C. Eight aliquots were collected for each sample at each time point.

For quantification of exatecan, a 20 μl sample was added to a polypropylene autosampler vial. 20 μl PPT-IS (acetonitrile (ACN):H_2_O (50:50) + 0.5% formaldehyde (FA) containing 1000 ng/mL internal standard) and 20 μl diluent (ACN:H2O (50:50) + 0.5% FA) was added to each sample. Followed by addition of 120 μl of ACN + 5% FA. The vials capped and vortexed for 2 min. The samples were centrifuged for 5–10 min at 3700 rpm then analyzed via liquid chromatography tandem mass spectrometry (LC–MS/MS).

### ELISA measurement of peptide plasma and tissue concentrations

96-well plates were coated with 100 μl/well of 0.1 μM BSA-labeled peptide prepared in 0.2 M carbonate-bicarbonate buffer, pH 9.4 and incubated overnight at 4°C. Plates were washed 4× with an ELISA wash buffer (PBS + 0.05% Tween 20), incubated for 2 h at room temperature with Blocking Buffer (PBS + 5% dry milk + 0.05% Tween 20) (300 μl/well) and washed again 4× with ELISA wash buffer. Concurrently, 2× CBX-12 standards (in respective tissue matrix) or sample plasma, tumor homogenates or bone marrow samples diluted with antibody diluent (PBS + 2% dry milk + 0.05% Tween 20), were pre-incubated with 1–10 ng/mL of 9H6.B3, a primary antibody specific for the CBX-12 peptide, for 30 min at room temperature. Pre-incubated samples were added to pre-coated, pre-blocked assay plates at 100 μl/ well and incubated for 1 h at room temperature. Plates were washed 4× with ELISA wash buffer and incubated with 100 μl/well of a secondary goat anti-mouse IgG HRP antibody (1:5000 in antibody diluent) for 1 h at room temperature. Plates were washed 4× with ELISA wash buffer and incubated with 100 μl/well of SuperSignal substrate at room temperature with gentle shaking for 1 min. Luminescence was read from the plate on a BioTek Cytation 5 plate reader.

### Biophysical measurements of CBX-12 interaction with lipid bilayer of membrane

Large unilamellar vesicles were prepared by extrusion. 1-Palmitoyl-2-oleoyl-sn-glycero-3-phosphocholine (POPC, Avanti Polar Lipids, Inc.) dissolved in chloroform was desolvated in a rotary evaporator to create a phospholipid film and placed under high vacuum for 2 h. Lipids were then rehydrated in phosphate buffer (pH 8) and extruded through membranes with pore size of 50 nm.

Absorption spectra of CBX-12 (20, 30 and 40 μM) in methanol were measured in a cuvette with 1 cm path length using a Genesys 10S UV–Vis (Thermo Scientific) spectrophotometer. The linear response in increase of absorbance was observed with increase of concentration. Steady-state fluorescence measurements were performed using a PC1 spectrofluorometer (ISS, Inc.). Circular dichroism (CD) and pH-dependence measurements were performed using a MOS-450 spectrometer (Biologic, Inc.). Tryptophan fluorescence spectra of pHLIP peptide and exatecan fluorescence were recorded with step of 1 nm at excitation wavelengths of 280, 295 or 360 nm. Excitation and emission polarizers were set to 54.7° and 0.0°, respectively. CD spectra were recorded from 210 to 260 nm with step of 1 nm. The concentration of CBX-12 and POPC were 7 μM and 1.4 mM in fluorescence and CD experiment, respectively.

The pH-dependent insertion of the peptide into the lipid bilayer of liposomes was studied by monitoring the changes in the molar ellipticity at 227 nm as a function of pH. After the addition of aliquots of HCl to reduce pH from pH8 to pH4, and aliquots of NaOH to raise pH from pH8 to pH9, the pHs of solutions containing 7 μM CBX-12 and 1.4 mM POPC liposomes were measured using an Orion PerHecT ROSS Combination pH Micro Electrode and an Orion Dual Star pH and ISE Benchtop Meter before and after each spectrum measurement to ensure that equilibrium is achieved. Millidegree at 227 nm were plotted as a function of pH in a normalized form from 0 to 1. The pH-dependence was fit with the Henderson-Hasselbach equation to determine the cooperativity ($n$) and the mid-point ($pK$) of a transition:

$$\begin{equation*}Normalized\;CD\;signal\; = \frac{1}{{1 + {{10}^{n\left( {pH - pK} \right)}}}}\;\end{equation*}$$

Fluorescence kinetics were measured using a SFM-3000 mixing system (Bio-Logic Science Instruments) in combination with the MOS-450 spectrometer with temperature control set to 25.0°C. All samples were degassed before measurements to minimize air bubbles in the samples. CBX-12 (14 μM) and POPC (2.8 mM) samples were incubated to reach equilibrium, when most of the peptide is associated with liposome lipid bilayers. To follow peptide insertion, equal volumes of peptide-POPC solution and HCl were fast mixed (5-ms dead time) to lower the pH from pH 8 to pH 4. To monitor fluorescence intensity changes of the peptide and exatecan during peptide insertion the emission signal was observed through a cut off 320 nm filter at an excitation of 295 nm.

All biophysics data were fit to the appropriate equations by nonlinear least squares curve fitting procedures employing the Levenberg Marquardt algorithm using Origin 8.5.

### Multimer experiments

CBX-12 was dissolved in DMSO (1 mg/μl), diluted in 5% mannitol citrate buffer, pH 10 (to concentration of 6.8 mg/ml) and further diluted in PBS pH7.4 containing physiological concentrations of Mg^2+^ and Ca^2+^ ions to obtain 88, 44 and 4.4 μM concentrations of CBX-12. The fluorescence and CD spectra were measured after preparation of samples and next day after dilutions.

### Xenograft experiments

All animal studies were approved by the Institutional Animal Use and Care Committee and performed in accordance with the Guide for the Care and Use of Laboratory Animals. All cell lines were maintained in RPMI media supplemented with 10% fetal bovine serum, 50 IU/ml Penicillin, 50 μg/mL Streptomycin, 2 mM Glutamax at 37°C in a humidified atmosphere with 5% CO_2,_ with the exception of the JIMT-1 model, which was grown in DMEM media supplemented with 10% fetal bovine serum. The cells were subcultured twice weekly and harvested during exponential growth for tumor inoculation. Mice were dosed as described below for each specific model.

### Compound administration

Intraperitoneal doses of 2.5, 5, 10 or 20 mg/kg CBX-12 (0.65, 1.3, 2.6 or 5.2 μmol/kg, respectively) or 1.15 or 2.3 mg/kg exatecan (2.6 or 5.2 μmol/kg, respectively) were prepared by diluting 0.1mg/μl DMSO stocks in 5% mannitol in citrate buffer. Compound was administered each day of dosing at a volume of 12 mL/kg (300 μl per 25 g mouse).

### Xenograft tumor growth measurements

Xenograft tumors were measured by calipers and volume was calculated using the equation for ellipsoid volume: Volume = π/6 × (length) × (width)^2^.

### Statistical analysis

Analysis of variance (ANOVA) was used to test for significant differences between groups. Post-hoc Bonferroni multiple comparison test analysis was used to determine significant differences among means. All statistical analysis was accomplished using Graph Pad Prism 8.2.0 software.

### HCT116 xenografts

Six-week-old female athymic nude *Foxn^nu^* mice were obtained from Taconic Labs (Cat# NCRNU-F). Each mouse was inoculated subcutaneously with HCT116 tumor cells (2.5 × 10^6^) in 0.1 mL of PBS with Matrigel (1:1). The tumors were then grown to a mean size of approximately 100–200 mm^3^ and the mice were then split into groups and treated as detailed in Table 1.

**Table 1.** Information on each group of animals for HCT116 xenograft experiments

Group	Treatment	Dose	Dosing schedule	Administration route	Number of mice
1	Vehicle (5% mannitol in citrate buffer)	NA	QDx4/wk x 3	i.p.	8
2	CBX-12	10 mg/kg	QDx4/wk x 3	i.p.	8
3	CBX-12	20 mg/kg	QDx4/wk x 3	i.p.	8
4	exatecan	1.15 mg/kg	QDx4/wk x 3	i.p.	8
5	exatecan	2.3 mg/kg	QDx4/wk x 3	i.p.	8
1	Vehicle (5% mannitol in citrate buffer)	NA	QDx4	i.p.	8
2	CBX-12	5 mg/kg	QDx4	i.p.	8
3	CBX-12	10 mg/kg	QDx4	i.p.	8
4	CBX-12	20 mg/kg	QDx4	i.p.	8
5	CBX-12	40 mg/kg	QDx4	i.p.	8
6	CBX-12	80 mg/kg	QDx4	i.p.	8

### MDA-MB-231 xenografts

Three to four-week-old female athymic nude *Foxn^nu^* mice were obtained from Envigo Labs. Each mouse was inoculated subcutaneously with MDA-MB-231 tumor cells (2 × 10^6^) in 0.1 mL of PBS with Matrigel (1:1). The tumors were then grown to a mean size of approximately 50–100 mm^3^ and the mice were then split into groups and treated as detailed in Table 2.

**Table 2.** Information on each group of animals for MDA-MB-231 xenograft experiments

Group	Treatment	Dose	Dosing schedule	Administration route	Number of mice
1	Vehicle (5% mannitol in citrate buffer)	NA	NA	i.p.	8
2	CBX-12	5 mg/kg	QDx4/wk x 3	i.p.	9
3	CBX-12	10 mg/kg	QDx4/wk x 3	i.p.	9
4	CBX-12	20 mg/kg	QDx4/wk x 3	i.p.	9
1	None	NA	NA	NA	9
2	Talazoparib	0.33 mg/kg	QDx15	p.o.	9
3	CBX-12	5 mg/kg	QDx4/wk x 3	i.p.	10
4	Talazoparib CBX-12	0.33 mg/kg 5 mg/kg	QDx15 QDx4/wk x 3	p.o. i.p.	8

### JIMT-1 xenograft

Five to six-week-old female NOD.SCID mice were obtained from Beijing Anikeeper Biotech Co., Ltd (Beijing, China). Each mouse was inoculated subcutaneously with JIMT-1 tumor cells (5 × 10^6^) in 0.1 mL of PBS with Matrigel (1:1). The tumors were then grown to a mean size of approximately 100 mm^3^ and the mice were then split into groups and treated as detailed in Table 3.

**Table 3.** Information on each group of animals for JIMT-1 xenograft experiments

Group	Treatment	Dose	Dosing schedule	Administration route	Number of mice
1	Vehicle (5% mannitol in citrate buffer)	NA	QDx4/wk x 3	i.p.	8
2	CBX-12	10 mg/kg	QDx4/wk x 3	i.p.	8
3	CBX-12	20 mg/kg	QDx4/wk x 3	i.p.	8

### MKN45 xenograft

Six-week-old female athymic nude *Foxn^nu^* mice were obtained from Taconic Labs (Cat# NCRNU-F). Each mouse was inoculated subcutaneously with MKN45 tumor cells (2 × 10^6^) in 0.1 mL of PBS with Matrigel (1:1). The tumors were then grown to a mean size of approximately 100–200 mm^3^ and the mice were then split into groups and treated as detailed in Table 4.

**Table 4.** Information on each group of animals for MKN45 xenograft experiments

Group	Treatment	Dose	Dosing schedule	Administration route	Number of mice
1	Vehicle (5% mannitol in citrate buffer)	NA	QDx4/wk x 2	i.p.	8
2	CBX-12	2.5 mg/kg	QDx4/wk x 2	i.p.	8
3	CBX-12	5 mg/kg	QDx4/wk x 2	i.p.	8
4	CBX-12	10 mg/kg	QDx4/wk x 2	i.p.	8
5	CBX-12	20 mg/kg	QDx4/wk x 2	i.p.	8
6	exatecan	1.15 mg/kg	QDx4/wk x 2	i.p.	8
7	exatecan	2.3 mg/kg	QDx4/wk x 2	i.p.	8

### Bone marrow collection

Following tumor collection, mice were euthanized by cervical dislocation under anesthesia in accordance with the American Veterinary Medical Association (AVMA) Guidelines for the Euthanasia of Animals. Femurs were removed, and bone marrow was extruded into 50 mL conical tubes by flushing the bones with a 23-gauge needle fitted on a 5cc syringe containing PBS + 2% fetal bovine serum (FBS). Bone marrow was homogenized by gentle pipetting and filtered through 100 μm nylon mesh filters and cells were pelleted by centrifugation at 1200 rpm for 5 min at 4°C. Red blood cells were lysed with 3 mL of lysis buffer for 2 min at room temperature. PBS was added to a volume of 25 mL and cells were re-pelleted by centrifugation as described above. Cell pellets were suspended in 5 mL of PBS and cell count was assessed by trypan blue exclusion on a TC-20 cell counter (BioRad).

### Xenograft tumor collection

At 6 h after compound administration on day 4, mice were anesthetized via continuous inhalation with isoflurane administered in 2% oxygen and were euthanized by cervical dislocation under anesthesia in accordance with the American Veterinary Medical Association (AVMA) Guidelines for the Euthanasia of Animals. Xenograft tumors were removed, weighed, and cut into smaller pieces with a scalpel blade. Random 100 mg tumor samples were collected in bead homogenization tubes, snap frozen in liquid nitrogen, and stored at -80°C until processed for measurement of CBX-12 concentrations.

### TOP1 assessments

#### Bone marrow collection for TOP1

Mouse bone marrow cells were isolated as described above.

#### Tumor collection for TOP1

Mice were anesthetized via continuous inhalation with isoflurane administered in 2% oxygen and were euthanized by cervical dislocation under anesthesia in accordance with the American Veterinary Medical Association (AVMA) Guidelines for the Euthanasia of Animals. Xenograft tumors were excised, weighed, and minced then were digested to single cells with an enzyme mix (Miltenyi; #130-095-929) for 40 min shaking at 37°C. Enzymes were inactivated by adding equal volume of DMEM^+^. Digested cells were sequentially filtered through 70 μm then 40 μm filters and centrifuged at 700 rpm for 7 min at 4°C. Cells were washed with PBS, RBCs were lysed, and cell count was assessed by trypan blue exclusion.

#### *In vitro* HCT116 cell dosing for TOP1

HCT116 cells were plated at 2.5 × 10^5^/mL in 2 mL DMEM^+^ in six-well plates. Cells were allowed to attach overnight in 37°C incubator before compound addition. Exatecan, CBX-12, or vehicle control were added to wells for final concentrations of 1000 to 0.46 nM (using 3-fold dilutions) for 24 h. Cells were scraped, washed with PBS and counted.

#### Ex vivo bone marrow cell dosing for TOP1

Naïve Sprague-Dawley rat bone marrow cells were plated at 1 × 10^6^/ml in 2 mL of RPMI^+^ in six-well plates. Cells were allowed to incubate for 3 h at 37°C before compound addition. Exatecan, CBX-12 or vehicle control were added to wells for final concentrations of 1000 to 0.46nM (using 3-fold dilutions) for 24 h. Cells were harvested, washed with PBS and counted.

#### TOP1 FACS assay

Tumor and bone marrow cells collected 24 h after dosing were permeabilized and fixed for 20 min on ice in the dark (Cytofix/Cytoperm Becton Dickinson; #554714), then washed 2× with Perm Wash. Cells were stained with rabbit anti-topoisomerase 1 antibody (Abcam; #109374) at 1 μg/ml on ice in the dark for 30 min and washed 2×, followed by staining with secondary antibody goat anti-rabbit IgG AF647 (Jackson ImmunoResearch; #111-605-144 diluted 1:1000) on ice in dark for 30 min. After a final 2× wash, the reduction of topoisomerase I (TOP1) protein levels was evaluated by collecting 10,000 (tumor) or 30,000 (BM) cells and determining the number of TOP1 positive stained cells on a BD Accuri™ flow cytometer.

### γH2AX assay

Tumors were collected as detailed above, and FFPE tumor slides were created for IF staining. Antigen retrieval was performed using a heat-induced epitope retrieval (HIER) method, with TRIS-EDTA pH 9.0. Staining with primary antibody (mouse anti-human γH2AX; 1:500, Invitrogen) followed by secondary antibody (Goat anti-mouse Ax594; 1:1000, Invitrogen) was then performed. Images were obtained with a Keyence imager. Data analysis was performed in ImageJ and CellProfiler (16). Raincloud plots and heatmap were generated in R version 4.0.3 (17). Statistical analysis for γH2AX expression was performed using Kruskal–Wallis test, followed by Dunn's multiple comparison test (GraphPad Prism 9.1.0).

### Amnis internalization assay

Rucaparib-peptide conjugates bearing the same peptide as CBX-12 were synthesized with cleavable and uncleavable linkers. HCT116 tumor cells were incubated in vitro with 5 μM compound, or DMSO vehicle for the indicated time, washed 2× with PBS, then incubated for 5 min with Cell Mask deep red plasma membrane stain (Invitrogen; #C10046), washed 2× with PBS/2% fetal bovine sera. 3,000 events were acquired from each sample with an Amnis ImageStream X-MKII imaging flow cytometer (EMD Millipore). Data was analyzed using IDEAS software for compound co-localization on membrane and internalization.

### Cell viability and synergy assays

Microplate-based short-term viability and synergy assays were performed as described previously (18).

### Apoptosis dye

Female nude mice with HCT116 flank tumors were dosed i.p., QDx4/week for 2 weeks with either vehicle or 10 mg/kg CBX-12. For imaging, mice were administered a single i.p. 7.3 mg/kg dose of PSVue® 749 (Molecular Targeting Technologies, Inc.) immediately following the fourth dose of CBX-12 in either the first or second dosing cycle. At 48 h post-administration of PSVue® 749, whole body imaging of anesthetized mice was performed using the LI-COR PEARL Trilogy small animal imager to visualize and quantitate the distribution of the dye. Images were analyzed using Image Studio Software (version 5.2). At 72 h post-administration of PSVue® 749, mice were euthanized, and tumors removed and imaged whole and cut in half to view distribution of dye throughout the tumor.

### Rat plasma pharmacokinetics (PK)

Male Sprague Dawley rats underwent jugular vein cannulation and insertion of a vascular access button (VAB, Instech Labs Cat # VABR1B/22) at Envigo Labs prior to shipment. Magnetic, aluminum caps (Instech Labs Cat # Cat #VABRC) were used to protect the access port for the jugular catheters allowing the animals to be housed 3 per cage on Alpha-Dri bedding for 4–5 days prior to the study. Rats were administered a single 5 mg/kg intravenous dose (1.3 μmol/kg) of CBX-12 prepared in a vehicle of 5% mannitol in citrate buffer. Blood was collected from fed rats at 1, 2, 4, 8, 24 and 30 h following compound administration. Exatecan concentrations were determined in plasma by LC–MS/MS. CBX-12 concentrations were determined in plasma by an ELISA assay specific for the peptide.

### Histology

Duodenal tissue samples were fixed in 10% neutral buffered formalin, processed and embedded in paraffin. Tissue cross sections (5 μm) were mounted on positively charged microscope slides and stained with hematoxylin and eosin (H&E) (PolyScientific R&D, New York). Images were taken of tissue sections by standard light microscopy using an EVOS FL Cell Imaging System (Life Technologies) for histomorphological evaluation of intestinal inflammation.

## RESULTS

**Figure 1.**
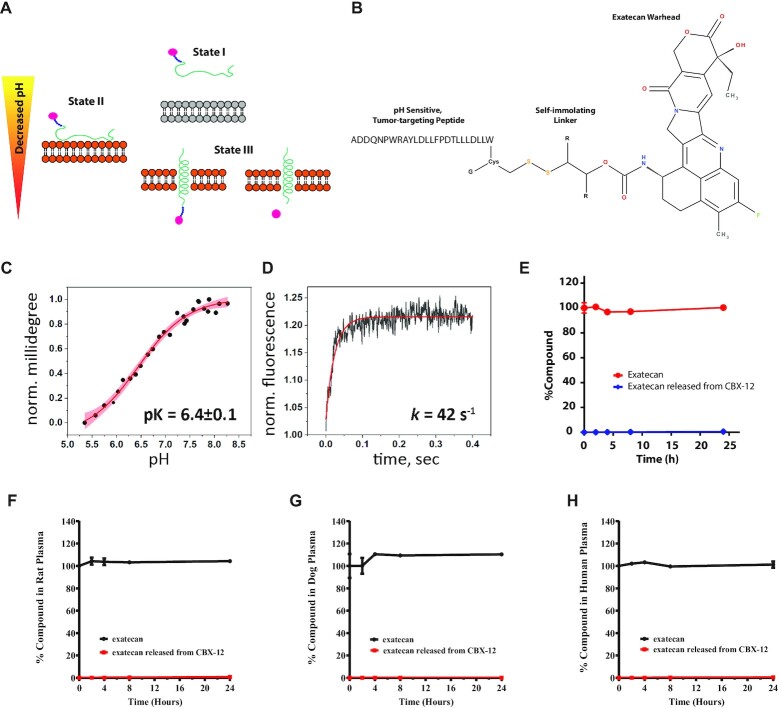
*In vitro* pH-dependent membrane insertion and stability of CBX-12 conjugates. (**A**) Schematic representation of a pHLIP conjugate interacting with and inserting across a lipid bilayer to deliver a cargo. (**B**) Design and structure of the CBX-12 conjugate. (**C**) The pH-dependent insertion of CBX-12 into bilayers of liposomes. (**D**) Kinetics of CBX-12 insertion into membrane of liposomes. (**E**) Time course of mouse (**E**) rat (**F**), dog (**G**) and human (**H**) plasma concentrations of exatecan after *in vitro* incubation with 10 μM CBX-12 or 10 μM unconjugated exatecan at 37°C. The percentage of exatecan released from CBX-12 or from unconjugated exatecan was calculated relative to unconjugated exatecan values at time = 0, *N* = 2.

As presented earlier, pHLIP conjugates directionally insert across cancer cell membranes in low pH conditions to deliver C-terminal warheads across the membrane with subsequent intracellular release of the agent, and a schematic of this process is shown in Figure 1A (14). The structure of CBX-12 consists of exatecan conjugated to a pHLIP variant via a self- immolating, glutathione sensitive linker (Figure 1B). CBX-12 undergoes a structural change and inserts directionally across the lipid bilayer of membranes in a pH-dependent manner (Figure 1C and Supplementary Figure S1). The midpoint of the transition for CBX-12 insertion in a membrane in synthetic liposomes is at pH 6.4. Since the rate constant of CBX-12 insertion into membrane is high (approximately 42s^−1^), insertion into tumor cells will occur even under conditions of fast blood flow and limited exposure time of CBX-12 to acidic cancer cells in vitro (Figure 1D). Further biophysical characterization of CBX-12 in solution by fluorescence and CD spectra established that CBX-12 is most likely in a monomeric form at concentrations of 4–5 μM, while forming oligomers at the higher concentrations of 44 and 88 μM (Supplementary Figure S2). The obtained results are in good agreement with previously published data showing that the pHLIP peptide by itself is monomeric at concentrations of 7 μM and below (19). Importantly, our previously published data indicate that even at high concentrations (up to 300 μM) the pHLIP peptide does not form oligomers larger than tetramers, as established by small-angle X-ray scattering (SAXS) (20). Thus, we expect that CBX-12 will exist as monomers when injected into humans and diluted in the total blood volume, and therefore that the interactions of the pHLIP peptides with the membranes of acidic cancer cells will occur as single molecular insertion events.

**Figure 2.**
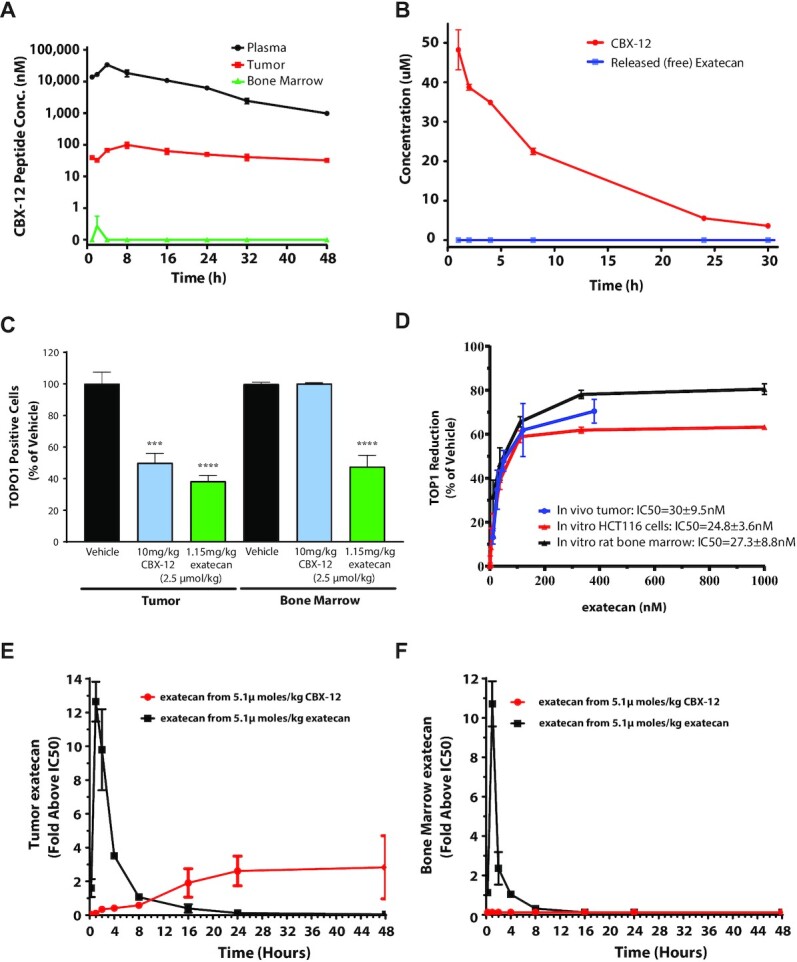
CBX-12 demonstrates tumor selective pharmacokinetics/pharmacodynamics *in vivo*. (**A**) Time course of CBX-12 peptide levels in plasma, HCT116 tumors, and bone marrow after a single 10 mg/kg dose of CBX-12 in mice. (**B**) Time course of plasma levels of CBX-12 peptide and unconjugated exatecan derived from CBX-12 after a single 5 mg/kg dose of CBX-12 in rats. Data are represented as mean ± SEM, N = 3-4 biological replicates for (A) and (B). (**C**) Number of TOP1 positive cells in HCT116 xenografted tumor and bone marrow from mice dosed with 2.5 μmol/kg of either CBX-12 or unconjugated exatecan for 4 days. Samples were taken 24 h post the last dose and assessed by FACS. (**D**) Levels of exatecan as assessed by LC/MS versus TOP1 protein reduction as assessed by FACS in xenografted HCT116 tumors of mice dosed for 4 days with CBX-12 *in vivo* (blue line), HCT116 cells treated with unconjugated exatecan for 24 h *in vitro* (red line), and rat bone marrow cells treated with of unconjugated exatecan for 24 h *in vitro* (black line), with a range of drug doses. (**E**) Time course of levels of exatecan assessed by LC/MS in HCT116 xenografted tumor of mice after dosing 5.1 μmol/kg of either CBX-12 (red line) or unconjugated exatecan (black line), and (**F**) the same experiment performed in bone marrow. Exatecan levels are expressed as increase above the *in vitro* IC50 of unconjugated exatecan against TOP1 protein levels in either HCT116 cells or rat bone marrow as established in (D).

*In vitro* plasma stability assays identified a CBX-12 peptide/linker conjugate combination which displayed robust linker stability and undetectable warhead release at 8 h and only minimal warhead release at 24 h, using mouse, rat, dog, and human plasma samples (Figure 1E–H). CBX-12 then was further characterized in a series of *in vivo* pharmacokinetics (PK) and pharmacodynamics (PD) studies. In these studies, we used the HER2- colon cancer model, HCT116, grown as flank xenografts. We demonstrated robust stability in the plasma of mice after a single injection, with rapid accumulation in tumor xenograft tissue but not in the bone marrow (Figure 2A). A similar plasma pharmacokinetics profile was observed in rats (Figure 2B).

To further validate that CBX-12 selectively targeted tumor and not healthy tissues, we developed a FACS-based functional assay to monitor the effect of TOP1 inhibition on TOP1 protein levels in tumor tissue and bone marrow, based on previously published methodology (21). In this assay, TOP1 inhibition directly correlates with loss of protein levels, such that the term, 50% inhibitory concentration (IC_50_) can be used for protein depletion. Using this assay, we demonstrated that TOP1 protein levels are decreased in both HCT116 flank tumor tissue and bone marrow following unconjugated exatecan administration, while CBX-12 selectively depletes TOP1 in the tumor, with no effect in the bone marrow (Figure 2C). We then performed bioanalytical measurements of exatecan in tumor versus bone marrow after dosing equimolar amounts of either CBX-12 or unconjugated exatecan *in vitro* and *in vivo*, and we compared these data to TOP1 protein levels in the corresponding samples. Following incubation for 24 h in HCT116 tumor cells and rat bone marrow cells *in vitro*, unconjugated exatecan inhibited similar dose-response curves for TOP1 protein levels, with IC50s of 24.8 nM and 27.3 nM, respectively (Figure 2D). We observed a similar dose-response curve for TOP1 protein inhibition in HCT116 tumor tissue after CBX-12 administration for 4 days *in vivo*, with an IC50 of 30 nM (Figure 2D). These data demonstrate a strong PK/PD relationship between exatecan exposure and TOP1 protein levels *in vitro* and *in vivo*. Using these data, we were able to accurately predict the levels of exatecan derived from CBX-12 in tumor, with dosing of CBX-12 resulting in both a dose-dependent increase in exatecan and decrease in TOP1 protein levels in the tumor (Supplementary Figure S3a). Subsequent studies revealed that a single dose of CBX-12 resulted in a gradual, time-dependent release in exatecan in the tumor, with values maintained 2- to 3-fold above the IC50 for TOP1 depletion *in vitro* (24.8 nM), which persisted for 48 h, while an equimolar dose of unconjugated exatecan induced a rapid spike in drug levels in the tumor, which rapidly decreased below the *in vitro* IC_50_ for TOP1 depletion within 8 h (Figure 2E). Unconjugated exatecan induced a similar pattern of exposure in the bone marrow, while no detectable levels of exatecan associated with CBX-12 injection were observed at any time point (Figure 2F). Together these data validate the ability of CBX-12 to selectively release active exatecan in tumor and not normal tissue.

**Figure 3.**
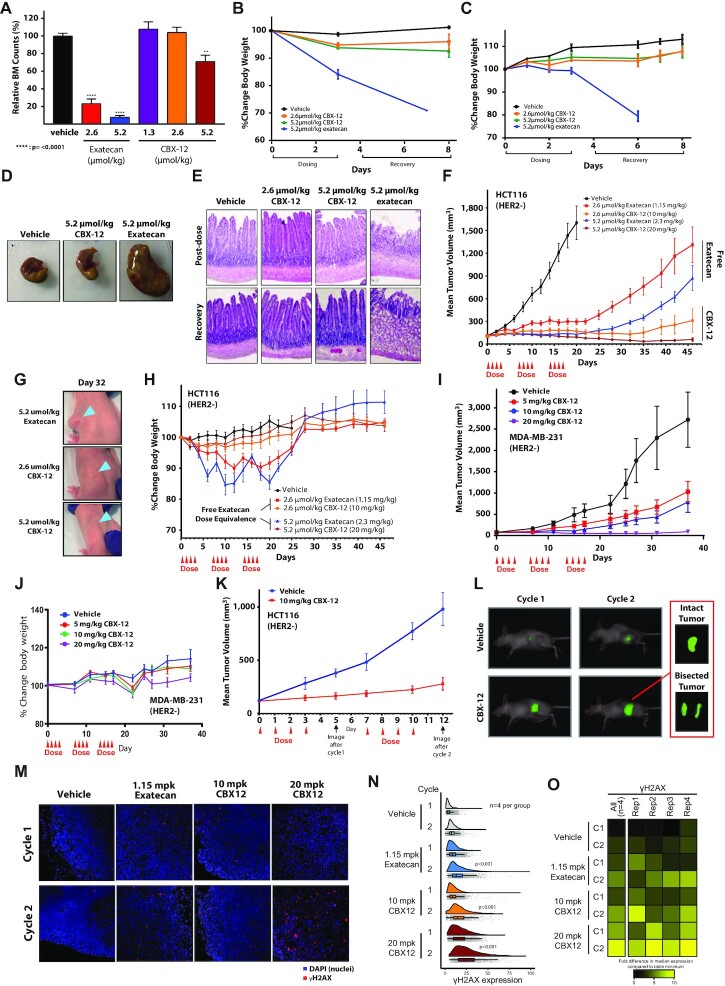
CBX-12 preferentially targets tumor xenografts over normal tissue and is highly effective against tumor xenografts as a monotherapy. (**A**) Bone marrow cell counts of mice dosed with either unconjugated exatecan or CBX-12 (*N* = 4). **** Indicates *P* < 0.0001 as determined by one-way ANOVA. (**B**) Percent change in body weight of mice dosed with either unconjugated exatecan or CBX-12. (**C**) Percent change in body weight of rats dosed with either unconjugated exatecan or CBX-12 at the indicated doses. Data are represented as mean ± SEM, *N* = 3–4 biological replicates for (B) and (C). (**D**) Dissected stomachs of mice dosed with either unconjugated exatecan or CBX-12. (**E**) Representative H&E staining of rat duodenums from mice treated with either unconjugated exatecan or CBX-12. Duodenum samples were harvested after the 4th dose (‘post-dose’) and after a 5 day recovery period. (**F**) Growth of HCT116 xenografts in nude mice dosed with either unconjugated exatecan or CBX-12 as indicated. Data are represented as mean ± SEM, *N* = 8 animals/arm. (**G**) Images of mice bearing HCT116 flank tumors at day 32 of the study in (F), arrows demonstrate location of xenograft. (**H**) Body weight over time of mice in the study in (F); note that 50% of the animals in the 2.3 mg/kg unconjugated exatecan group had to be euthanized before completion of the experiment because of severe morbidity due to drug toxicity. (**I**) Growth of MDA-MB-231 xenografts in nude mice dosed with CBX-12 as indicated. Data are represented as mean ± SEM, *N* = 8–9 mice/arm. (**J**) Body weight over time of mice in the study in (I). (**K**) Growth of HCT116 tumor xenografts in mice dosed with vehicle or CBX-12 which were imaged for apoptosis using the PSVue^®^ 749 apoptosis dye at the indicated time-points. Data are represented as mean ± SEM, *N* = 8 mice/arm. (**L**) Representative images demonstrate PSVue^®^ 749 signal distribution in a representative mouse from each dose group. Inset: *Ex vivo* bisected tumor of the indicated mouse 72 h after dye injection. (**M**) Immunofluorescence images (taken at 20×) of representative xenografts after one (top row) and two (bottom row) cycles treatment with vehicle, 1.15 mg/kg exatecan, 10 mg/kg CBX-12, and 20 mg/kg CBX-12. Blue and red denote DAPI and γH2AX, respectively. (**N**) Raincloud plots displaying single cell-level γH2AX expression (x-axis) in xenografts after different treatments (y-axis). Each group includes 4 xenograft replicates. Individual gray dots represent single cells. Vertical lines in boxplots are median expression levels and error bars represent 2 standard deviations. Treatments after the second cycle led to a significant higher γH2AX expression compared to the first cycle for exatecan (*P* < 0.001), 10 mg/kg CBX-12 (*P* < 0.001) and 20 mg/kg CBX-12 (*P* < 0.001; indicated in the corresponding raincloud plots). (**O**) Heatmap displays the fold change differences of the median γH2AX expression in single cells compared to table's minimum (replicate 3, vehicle treatment cycle 1). Left most column denotes all 4 replicates combined and the right 4 columns represent individual replicates. Rep = replicate, C = cycle.

We then sought to assess the extent to which treatment with CBX-12 can bypass the severe toxicities induced by dosing unconjugated exatecan. While unconjugated exatecan induces drastic bone marrow suppression, dosing an equimolar amount of CBX-12 maintains bone marrow counts at comparable levels to control animals (Figure 3A). Concordantly, significant and unrecoverable body weight reductions were consistently noted in animals treated with unconjugated exatecan, while minimal effects were noted with CBX-12 (Figure 3B, C). These effects of unconjugated exatecan on body weight correlated with significant GI toxicity both at the whole organ level (Figure 3D) and microscopically (Figure 3E). In contrast, animals dosed with CBX-12 did not display any detectable GI toxicity (Figure 3D, E).

Next, we assessed the efficacy of CBX-12 in tumor xenograft models *in vivo*. As the recently approved ADC DS-8201a is restricted to HER2+ cancers, we tested HER2- models to highlight the antigen independence of our tumor targeting approach. We first utilized the HER2- colon cancer model, HCT116 presented above. We confirmed an *in vitro* cell killing IC50 of 0.2 nM in HCT116 cells (data not shown), which is consistent with previously published reports demonstrating broad activity of the drug across multiple cell lines (22). As shown in Figure 3F, we observed a dose-dependent tumor killing in HCT116 flank xenografts with the administration of CBX-12 for three 4-day cycles. This response was notable for complete tumor regressions (Figure 3G) and superior efficacy and tolerability relative to dosing an equimolar amount of unconjugated exatecan (Figure 3F–H). For example, 50% of the animals in the 2.3 mg/kg unconjugated exatecan group had to be euthanized before completion of the experiment because of severe morbidity due to drug toxicity. We observed similar efficacy of CBX-12 in the HER2 negative breast cancer model, MDA-MB-231 (Figure 3I). In these experiments, we did not observe significant adverse effects on animal weights (Figure 3J). To further demonstrate that this efficacy was due to selective delivery of unconjugated exatecan to tumors by CBX-12, we demonstrated that the pHLIP peptide alone did not have any activity *in vivo* (Supplementary Figure S4), and that a non-cleavable control conjugate bearing a fluorescent warhead displayed no evidence of warhead entry from the cell membrane into the cytoplasm, which support our established mechanism of action of the peptide (Supplementary Figure S2g–i).

In another tumor growth delay study with the HCT116 model, we also assessed CBX-12-induced apoptosis in flank tumors after two cycles of treatment. These studies revealed a correlation between sustained suppression of tumor growth and homogenous apoptotic staining throughout the tumor (Figure 3K and L, respectively). We also demonstrated enhanced DNA damage in HCT116 flank tumors treated with unconjugated exatecan and CBX-12, as detected by γH2AX immunofluorescence (Figure 3M). These experiments were notable for greater levels of DNA damage with CBX-12 versus unconjugated exatecan (Figure 3N and O). Treatments after the second cycle led to a significantly higher γH2AX expression compared to the first cycle for exatecan (*P* < 0.001), 10 mg/kg CBX-12 (*P* < 0.001) and 20 mg/kg CBX-12 (*P* < 0.001). Treatment with 10 mg/kg CBX-12 resulted in a similar γH2AX expression as exatecan (cycle 1, *P* = 0.54; cycle 2, *P* = 0.22). However, 20 mg/kg CBX-12 treatment showed a significant increase in DNA damage compared to 10 mg/kg CBX-12 and exatecan after both cycles (*P* < 0.001), which suggests a dose–response effect. Collectively, these data highlight the robust selectivity of these conjugates to target tumor over normal tissue, with durable tumor growth suppression and minimal systemic toxicity.

**Figure 4.**
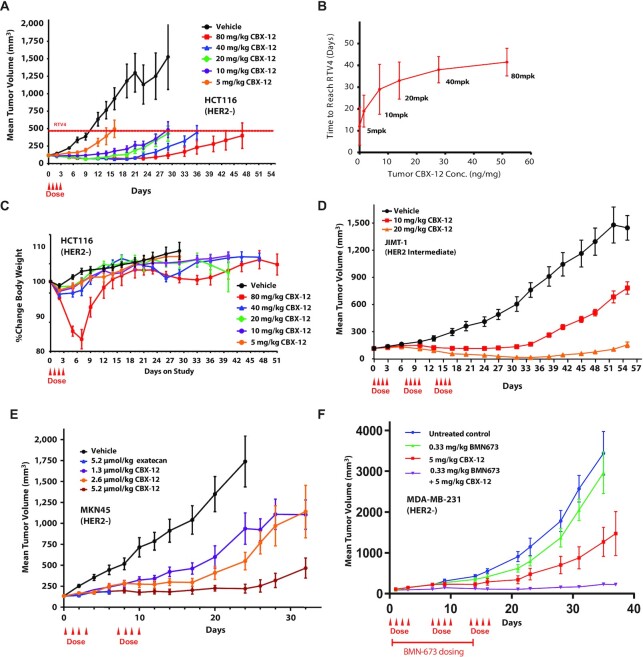
Anti-tumor efficacy with CBX-12 using alternative dosing regimens, additional tumor models, and in combination with the PARP inhibitor talazoparib. (**A**) Growth of HCT116 xenografts in nude mice dosed with CBX-12. The dashed line indicates the quadrupled initial tumor volume (RTV4). *N* = 8 mice/arm. (**B**) Comparison of time to reach RTV4 to intratumoral CBX-12 concentrations for HCT116 xenografts from (A) in mice treated with the indicated CBX-12 concentrations. (**C**) Body weight over time for mice in the study from (A). (**D**) Growth of JIMT-1 xenografts in SCID mice dosed with CBX-12. *N* = 8 mice/arm. (**E**) Growth of MKN45 xenografts in nude mice dosed with unconjugated exatecan or CBX-12. *N* = 8 mice/arm. (**F**) Growth of MDA-MB-231 xenografts in nude mice dosed with talazoparib (BMN673) or CBX-12 as single agents or in combination. Red line indicates talazoparib dosing. *N* = 8–10 mice/arm. All data are represented as mean ± SEM, and arrows represent when the doses of CBX-12 or exatecan were given.

Given the prolonged half-life of CBX-12 in the plasma and tumor tissue, and our findings indicating that CBX-12 is more efficacious than equimolar doses of unconjugated exatecan (Figure 3F), we also tested a range of alternative CBX-12 dosing regimens in the HCT116 flank model. We found that even a single cycle of CBX-12 infused at 10 mg/kg was sufficient for robust tumor cell killing, with both efficacy and intratumoral drug concentrations beginning to saturate at this 10 mg/kg dose (Figure 4A, B). In these experiments, we did not observe significant adverse effects on animal weights, except for recoverable weight loss in the very highest 80 mg/kg dosing regimen (Figure 4C).

We then demonstrated that CBX-12 was active in two additional flank tumor xenograft models, JIMT-1 (breast cancer, intermediate HER2 expression) and MKN45 (gastric cancer, HER2 negative), using either a two or three dosing cycle regimen as presented above. The JIMT-1 cells were obtained from a pleural metastasis in a patient who was clinically resistant to the HER2 targeting antibody, trastuzumab (23). DS-8201 has demonstrated only modest efficacy at the highest ADC doses in this model (24). In contrast, our data demonstrates robust anti-tumor activity in the JIMT-1 model, with near complete regression induced by three cycles of the 20 mg/kg dose, and with minimal effects on body weight (Figure 4D and Supplementary Figure S3b). Similarly, we observed extremely potent and durable anti-tumor activity in the MKN45 model, in this case with only two dosing cycles (Figure 4E and Supplementary Figure S3c). We also compared a dose of unconjugated exatecan that was equimolar to 20 mg/kg CBX-12 in the MKN45 flank model (2.3 mg/kg) in these experiments, and we found that this dose was not tolerated and was lethal in all animals in this cohort.

Finally, we sought to test whether CBX-12 could be combined with systemically administered DNA damage response (DDR) inhibitors. Previous studies have demonstrated synergistic interactions between TOP1 and DDR inhibitors, which has prompted great interest in testing these combinations in the clinic (25). In particular, the efficacy of PARP inhibitors as monotherapies have been limited to tumors with HR defects (HRD+), but it has been postulated that combinations with DNA damaging agents (including TOP1 inhibitors) would extend their activity to HRD- tumors (1). However, dose-limiting myelosuppression has been a major barrier to achieving treatment efficacy with these combinations, with up to 30–50-fold dose reductions required in clinical trials at the maximum tolerated dose (MTD) for these regimens (26–28). We hypothesized that a tumor-targeted exatecan would enable the use of DDR inhibitor-based combinations, thus extending the activity of DDR inhibitors to HRD- tumors. To this end, we tested the safety and efficacy of CBX-12 in combination with the potent PARP-trapping PARP inhibitor, talazoparib (29), in the (HRD-) MDA-MB-231 model. Of note, talazoparib has been shown to synergize with TOP1 inhibitors in pre-clinical studies (29,30), but the combination requires significant dose reductions in the clinic and at these limited doses has shown minimal efficacy in patients (31). Short-term viability assays confirmed the activity of unconjugated exatecan in vitro in MDA-MB-231 cells (with an IC50 of 0.21 nM), and we observed robust synergy when this drug was combined with talazoparib (Supplementary Figure S3e and f, respectively). As shown in Figure 4F, we observed robust tumor killing with a combination of CBX-12 at 5 mg/kg and talazoparib at a dose of 0.33 mg/kg *in vivo*, with minimal adverse effects on animal weights (Supplementary Figure S3d). In contrast, similar combinations of talazoparib with non-targeted versions of DNA damaging agents (in this concentration range) are poorly tolerated in animals *in vivo* (32,33). In addition, there is only modest anti-tumor activity when these agents are delivered separately as monotherapies in this model at doses equimolar to those used here (32,33).

## DISCUSSION

In conclusion, we have described a new approach for delivering the potent TOP1 inhibitor exatecan selectively to tumor cells in an antigen-independent manner via conjugation to a variant of pHLIP. The resulting CBX-12 conjugate displays pH selectivity, stability in plasma and selectivity to tumor cells versus healthy tissue. Importantly, CBX-12 displays extremely potent and durable anti-tumor activity in a variety of xenograft models with minimal to no side effects relative to the unconjugated exatecan warhead, equimolar amounts of which are poorly tolerated. As noted above, these conjugates leverage a variant of a pH-Low Insertion Peptide (pHLIP), which has been tested in numerous animal models previously without any evidence of significant toxicity associated with the peptide alone (reviewed in (14)). Consistent with these findings, we have not observed any significant toxicities with fluorescently tagged versions of the peptide, the data presented here with CBX-12 reveal no evidence of GI or bone marrow toxicity.

One limitation of this study is that we did not evaluate sequence variants of the pHLIP peptide or the requirement of the self-immolating linker for delivery and release of exatecan into tumor cells. However, we did demonstrate that the pHLIP peptide alone does not have any activity *in vivo* (Supplementary Figure S4). In addition, a non-cleavable control conjugate bearing a fluorescent warhead displayed no evidence of warhead release from the cell membrane into the cytoplasm, which supports the mechanism of action of the peptide conjugate (Supplementary Figure S2g–i). Nonetheless, future structure-activity relationships in the context of exatecan conjugation will likely shed additional insights into this interaction.

The significantly enhanced therapeutic window of CBX-12 relative to unconjugated exatecan enables the possibility of combinations that were previously too toxic in the clinic to use at efficacious doses, such as with DDR inhibitors. Because highly efficacious doses can be given with minimal toxicity, it also provides the basis to explore novel combinations such as with immunotherapies. Moreover, CBX-12 showcases a powerful approach to targeted tumor delivery that can be adapted to different potent small molecule warheads and used in an antigen agnostic manner in patients whose tumors cannot be targeted with current antibody-based technology. As such, CBX-12 has entered the clinic in 2021 to define a maximally tolerated dose with monitoring of safety, PK/PD, and preliminary efficacy (NCT04902872).

## Supplementary Material

zcab021_Supplemental_File
