# Correction to ‘Tumor-selective, antigen-independent delivery of a pH sensitive peptide-topoisomerase inhibitor conjugate suppresses tumor growth without systemic toxicity’

**DOI:** 10.1093/narcan/zcab047

**Published:** 2021-12-07

**Authors:** Sophia Gayle, Robert Aiello, Nalin Leelatian, Jason M Beckta, Jane Bechtold, Patricia Bourassa, Johanna Csengery, Robert J Maguire, Dan Marshall, Ranjini K Sundaram, Jinny Van Doorn, Kelli Jones, Hunter Moore, Lori Lopresti-Morrow, Timothy Paradis, Laurie Tylaska, Qing Zhang, Hannah Visca, Yana K Reshetnyak, Oleg A Andreev, Donald M Engelman, Peter M Glazer, Ranjit S Bindra, Vishwas M Paralkar

**Affiliations:** Cybrexa Therapeutics, New Haven, CT 06511, USA; Cybrexa Therapeutics, New Haven, CT 06511, USA; Department of Pathology, Yale University School of Medicine, New Haven, CT 06520, USA; Department of Therapeutic Radiology, Yale University School of Medicine, New Haven, CT 06520, USA; Cybrexa Therapeutics, New Haven, CT 06511, USA; Cybrexa Therapeutics, New Haven, CT 06511, USA; Cybrexa Therapeutics, New Haven, CT 06511, USA; Cybrexa Therapeutics, New Haven, CT 06511, USA; Cybrexa Therapeutics, New Haven, CT 06511, USA; Department of Therapeutic Radiology, Yale University School of Medicine, New Haven, CT 06520, USA; Department of Therapeutic Radiology, Yale University School of Medicine, New Haven, CT 06520, USA; Cybrexa Therapeutics, New Haven, CT 06511, USA; Cybrexa Therapeutics, New Haven, CT 06511, USA; Cybrexa Therapeutics, New Haven, CT 06511, USA; Cybrexa Therapeutics, New Haven, CT 06511, USA; Cybrexa Therapeutics, New Haven, CT 06511, USA; Cybrexa Therapeutics, New Haven, CT 06511, USA; Physics Department, University of Rhode Island, Kingston, RI 02881, USA; Physics Department, University of Rhode Island, Kingston, RI 02881, USA; Physics Department, University of Rhode Island, Kingston, RI 02881, USA; Department of Molecular Biophysics and Biochemistry, Yale University, New Haven, CT 06511, USA; Department of Therapeutic Radiology, Yale University School of Medicine, New Haven, CT 06520, USA; Department of Genetics, Yale University School of Medicine, New Haven, CT 06520, USA; Department of Pathology, Yale University School of Medicine, New Haven, CT 06520, USA; Department of Therapeutic Radiology, Yale University School of Medicine, New Haven, CT 06520, USA; Cybrexa Therapeutics, New Haven, CT 06511, USA

*NAR Cancer*, Volume 3, Issue 2, June 2021, zcab021, https://doi.org/10.1093/narcan/zcab021

The Authors wish to add a new source of Funding:

## FUNDING

NIH [R35CA197574 to P.M.G., R01CA215453 to R.S.B.]; Cybrexa Therapeutics. The work was partially supported by NIH/NIGMS [R01 GM073857] to O.A.A., Y.K.R. and D.M.E. Funding for open access charge: National Institutes of Health [R01CA215453, R35CA197574].

The published article has been updated.

