# Supplementary material for: Tumor-selective, antigen-independent delivery of a pH sensitive peptide-topoisomerase inhibitor conjugate suppresses tumor growth without systemic toxicity
Source: NAR Cancer. 2021 Jun 4;3(2):zcab021. doi: 10.1093/narcan/zcab021 (PMC8210154; doi:10.1093/narcan/zcab021)

Supplementary Figure 1. Biophysical characterization of CBX-12 interaction with a membrane lipid bilayer *in vitro*. Absorbance spectra of CBX-12 and exatecan in methanol (a); circular dichroism (b), fluorescence spectra excited at 295 nm (c) and at 360 nm (d) of CBX-12 measured in phosphate buffer at pH 8 in absence of POPC liposomes (state I, black lines) and at pH 8 in presence of POPC liposomes (state II, blue lines), and in the presence of POPC liposomes at pH 5 (state III, red lines); kinetics of fluorescence changes first 20 sec, (e) and 600 sec, (f) in response to pH drop reflecting CBX-12 insertion into membrane.

Supplementary Figure 2. Analysis of CBX12 multimer formation. CD (a) and fluorescence (excited at 295 nm) (b) spectra of different concentrations of CBX-12 are shown.

Supplementary Figure 3. Additional *in vivo* PK/PD, efficacy and safety data for CBX-12. (a) Relationship of intratumoral levels of unconjugated exatecan as determined by LC/MS to percent reduction of tumor TOP1 protein levels as assessed by FACS of HCT116 tumor cells of female athymic nude mice dosed with vehicle or 5, 10, 20, 40 or 80mg/kg CBX-12 once daily for 4 days. Tumor cells isolated from samples collected 4 hours after the dosing period. Data are expressed as means ± SEM (n=4; 5mg/kg=2). (b) Body weight of mice over time corresponding to the JIMT-1 study from Fig. 4d. SCID mice bearing JIMT-1 tumor xenografts were dosed with CBX-12 as indicated. N=8 mice/arm. (c) Body weight of mice over time corresponding to the MKN45 study from Fig. 4e. (d) Body weight of mice over time for the MDA-MB-231 study presented in Fig. 4F. (e) *In vitro* short-term viability assay testing exatecan in MDA-MB-231 cells. (f) Bliss synergy analysis *in vitro* for synergistic interactions between exatecan and BMN673 (talazoparib). (g) Representative Amnis images of the intrinsic fluorescence derived from rucaparib (green) relative to the plasma membrane (red) of HCT116 cells after incubation with either a cleavable or non-cleavable rucaparib peptide conjugate. (h) Quantitative assessments of the number of cells with colocalization of the rucaparib warhead with the cell membrane or (i) within the cytoplasm after incubation of either a cleavable or non-cleavable rucaparib peptide conjugate.

Supplementary Figure 4. Growth of HCT116 xenografts in nude mice dosed with 20 mg/kg of peptide alone compared to 20 mg/kg of CBX-12 for two cycles each.

Supplementary Figure 1

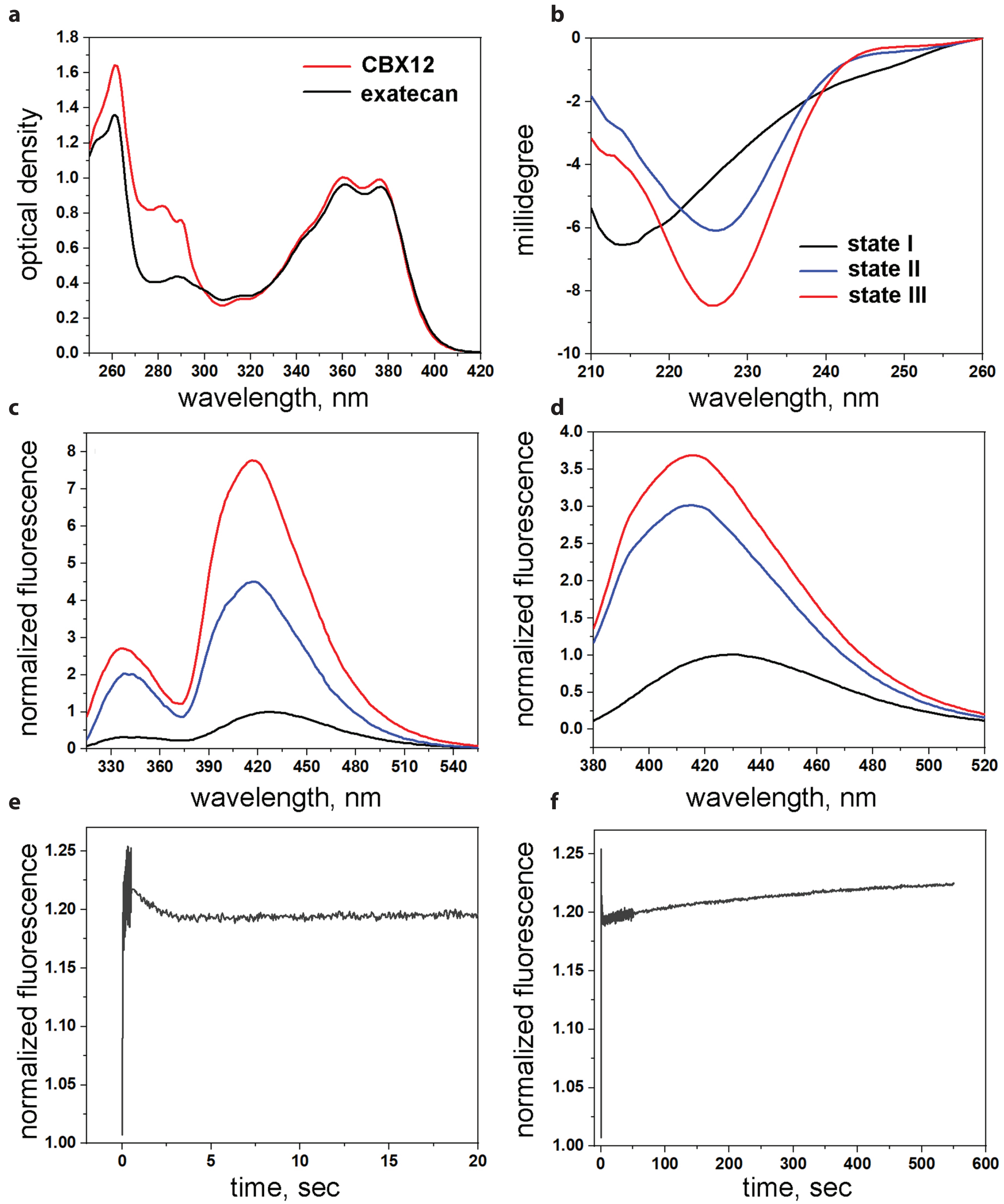

Supplementary Figure 2

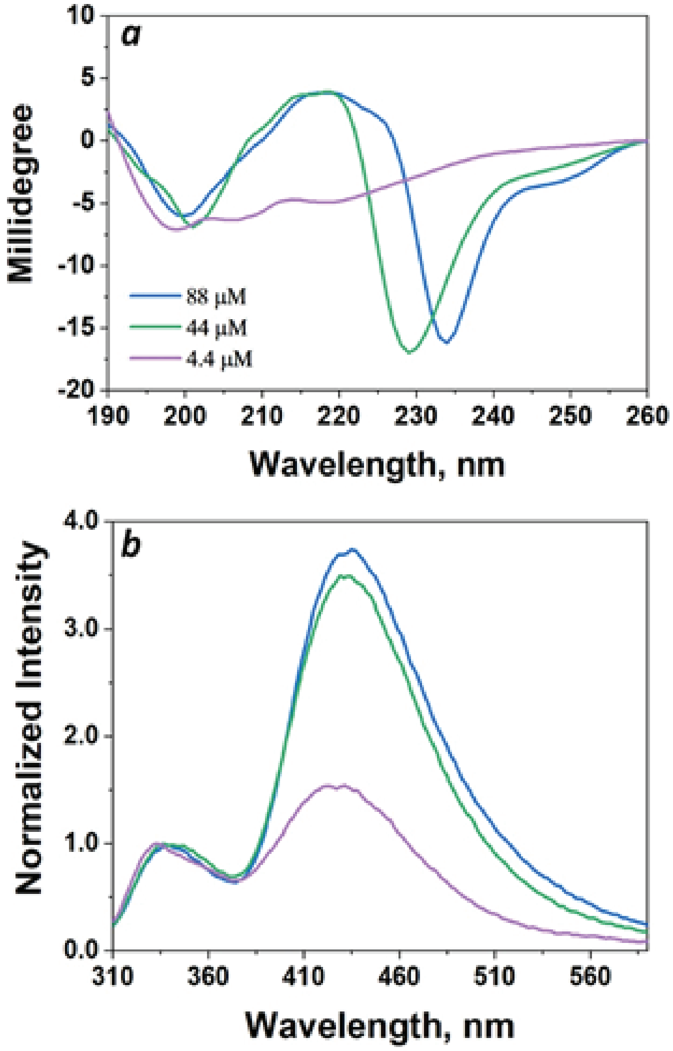

Supplementary Figure 3

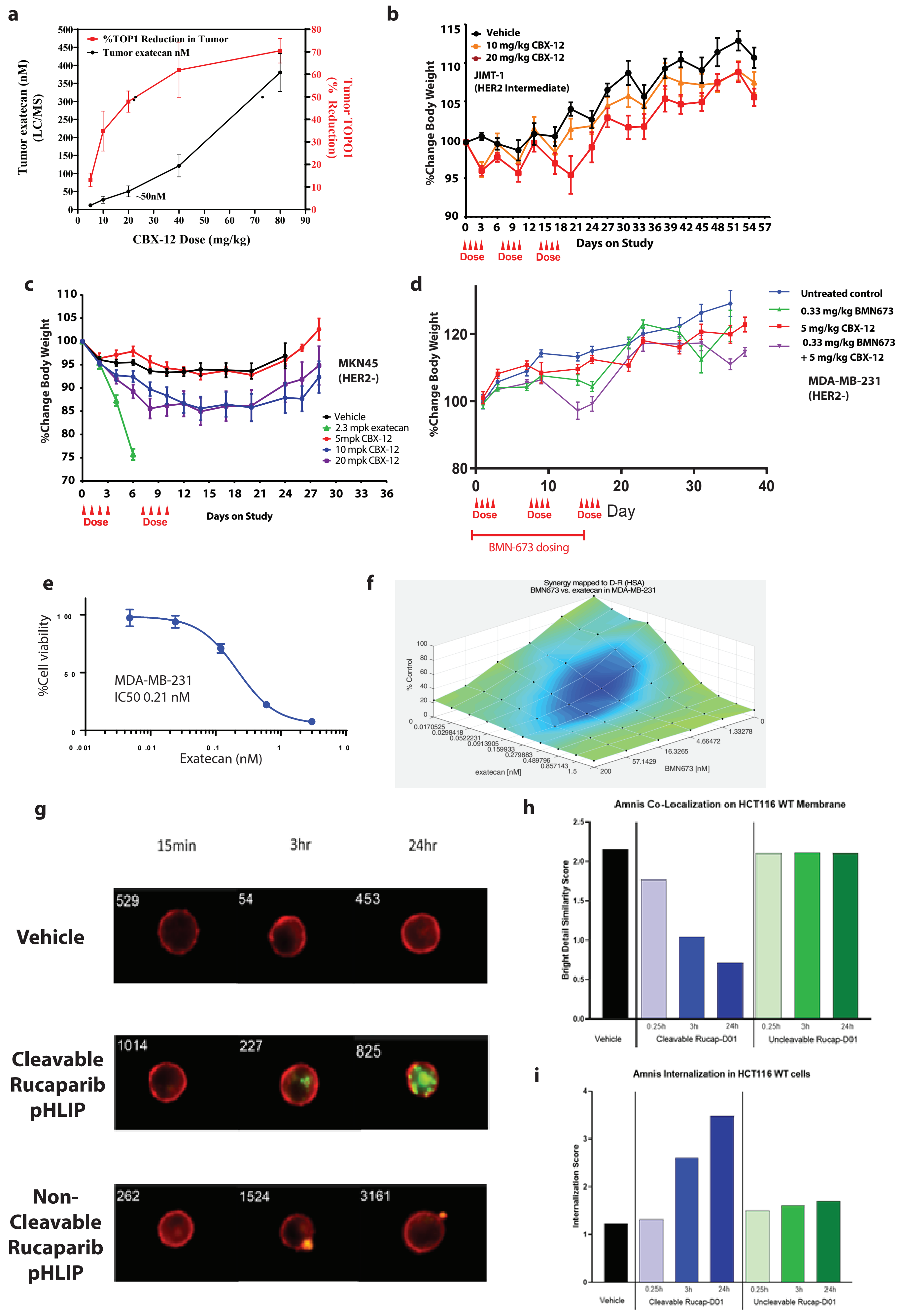

Supplementary Figure 4

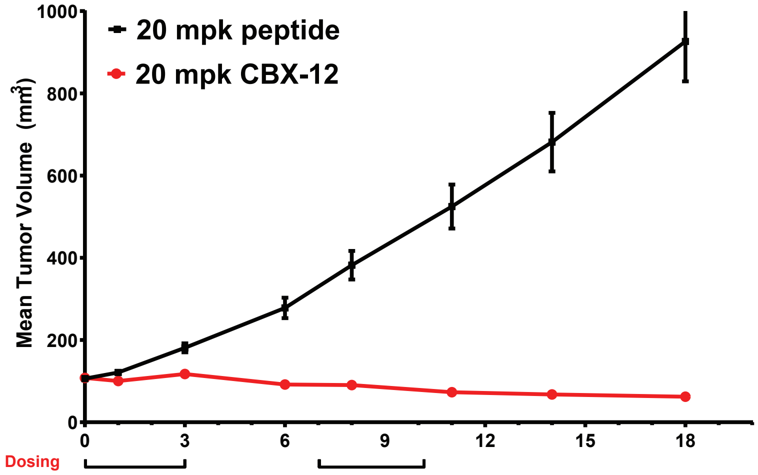

Supplement: zcab021_Supplemental_File [file zcab021_supplemental_file.pdf]
